# GeneKeyDB: A lightweight, gene-centric, relational database to support data mining environments

**DOI:** 10.1186/1471-2105-6-72

**Published:** 2005-03-24

**Authors:** SA Kirov, X Peng, E Baker, D Schmoyer, B Zhang, J Snoddy

**Affiliations:** 1 Graduate School for Genome Science and Technology, Oak Ridge National Laboratory-University of Tennessee, Oak Ridge, USA; 2 Life Sciences Division, Oak Ridge National Laboratory, Oak Ridge, USA; 3 Department of Engineering and Computer Science, Baylor University, Waco, USA

## Abstract

**Background:**

The analysis of biological data is greatly enhanced by existing or emerging databases. Most existing databases, with few exceptions are not designed to easily support large scale computational analysis, but rather offer exclusively a web interface to the resource. We have recognized the growing need for a database which can be used successfully as a backend to computational analysis tools and pipelines. Such database should be sufficiently versatile to allow easy system integration.

**Results:**

GeneKeyDB is a gene-centered relational database developed to enhance data mining in biological data sets. The system provides an underlying data layer for computational analysis tools and visualization tools. GeneKeyDB relies primarily on existing database identifiers derived from community databases (NCBI, GO, Ensembl, et al.) as well as the known relationships among those identifiers. It is a lightweight, portable, and extensible platform for integration with computational tools and analysis environments.

**Conclusion:**

GeneKeyDB can enable analysis tools and users to manipulate the intersections, unions, and differences among different data sets.

## Background

As we move toward large-scale research into complex molecular and cellular networks, the research community will need to develop new interfaces to complex data sets. Existing databases and interfaces, such as those at EBI and NCBI[1,2], often use sequence records as the central organizing unit. A database organization around a genome sequence record, for example, might be ideal for the purpose of a genome analysis, while the analysis of biological networks would be better organized around genes and gene products. LocusLink [3] (soon to be replaced by Entrez Gene [4]) is an example of a resource that adapts the more suitable gene-centric view. While having excellent user interfaces (UIs), LocusLink does not provide robust application programming interfaces (APIs). Even though an API could use web interface or a flat file database, this would make the analysis tool unacceptably slow. In particular, APIs are needed for computers to process the sets of the genes and gene products that are found in these biological networks. Both computational tools and advanced data mining environments need to use these APIs to access and manipulate large, diverse, and intersecting sets of data. EBI's EnsMART [5] is a resource that permits a comparable manipulation of data about sets of genes and provides an API along with the UI. The database, however, is somewhat difficult to store locally due to its large size and complexity. Another database that is to some extent similar with respect to the design is the DRAGON database[6]. We have developed GeneKeyDB, a relational database, in an attempt to address these issues. A schematic comparison of these databases and GeneKeyDB can be seen in Table 1.

An interesting alternative to the above mentioned databases is BioMART[7]. This is not a database in the conventional sense (though the underlying data can also be downloaded). It extracts and integrates data from several sources, creating customized database. While this approach is very powerful, not all data present in GeneKeyDB is available from BioMART sources (for example CGAP expression data or Homologene). Still BioMART requires a human intervention to retrieve the customized database through a web interface, where GeneKeyDB can be updated entirely through scripts.

The development of GeneKeyDB is motivated by a desire to have a smaller-sized database that could tightly interoperate with different local computational tools and local data sets. While providing support for different data mining tools, the database may remain lightweight, by just storing the keys (database identifiers) of objects, some important attributes, and the relationships among the objects. The database does not actually need to store large objects, like sequence records, as long any tool built on top of GeneKeyDB can retrieve the subset of the objects of interest on demand from other sources – EBI, NCBI or local storage. The system allows us to create centrally shared functions to manipulate biological data sets. Each analysis tool, analysis pipeline, or computer can rely on these core functions and data found in GeneKeyDB.

## Construction and content

**Figure 1:**
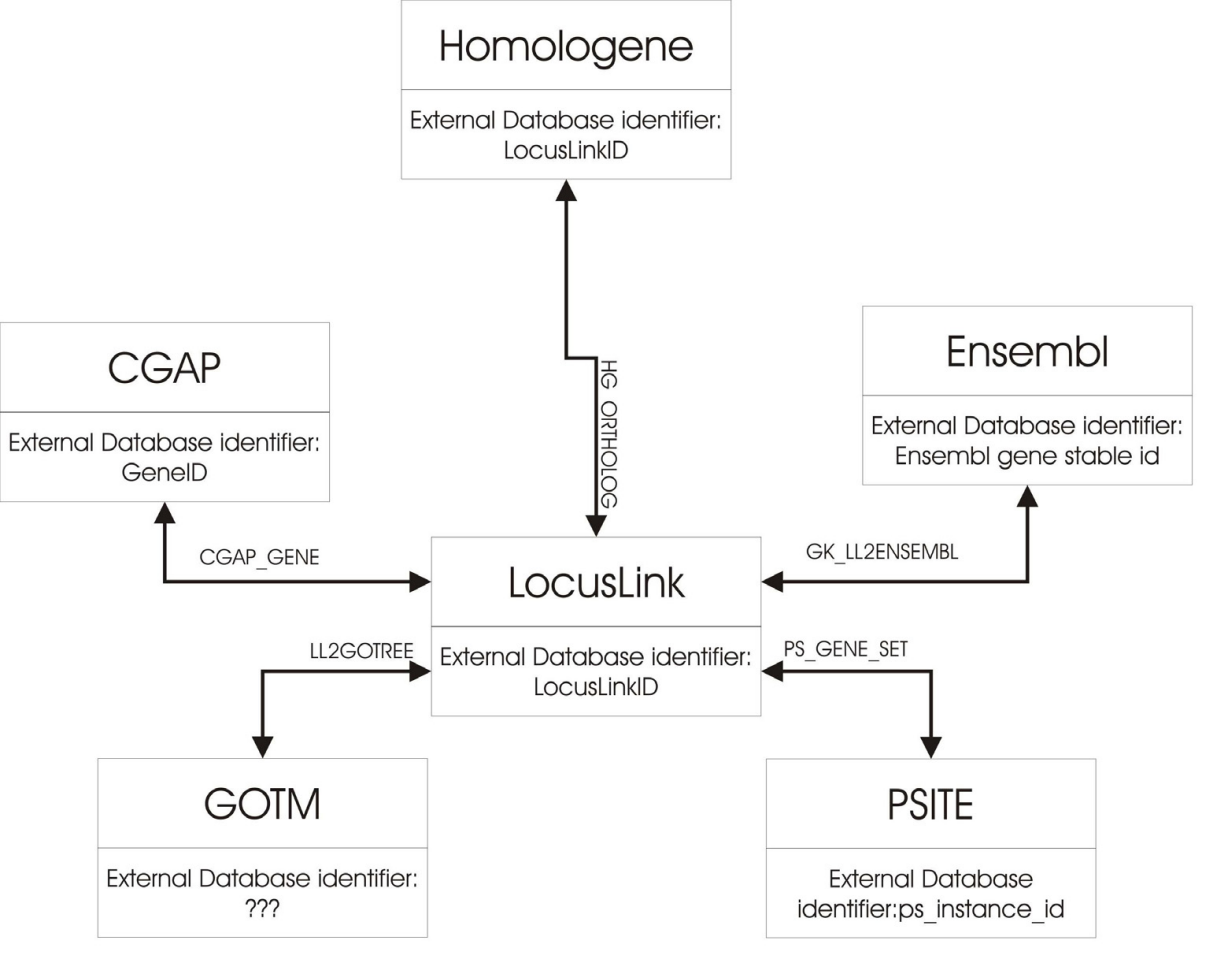
GeneKeyDB sub-modules, external database identifiers and connecting tables. The connecting tables may convert between the central key and another unique key used throughout the sub-module and are shown next to the connector lines.

GeneKeyDB consists of several sub-modules (Figure 1), corresponding to the represented data sources or supported services- LocusLink, EnsEMBL, HomoloGene, GoTreeMachine (GOTM), MGI comparative map and CGAP tables [1,2,8-10]. Additional modules can be easily added through the central key of the database. For example we are developing a Cis-Regulatory Elements database called PSITE (manuscript in preparation) which is already integrated to GeneKeyDB through LocusLink ids (Entrez Gene identifiers). In a similar way a supporting GOTM database module has been created previously [10]. Consistency between the database sub-modules is achieved through the creation of novel joining relations. The overall effect is a schema where no data connection is more than two relational tables apart. Summary of the data, provided by each source can be seen in Table 2.

**Figure 2:**
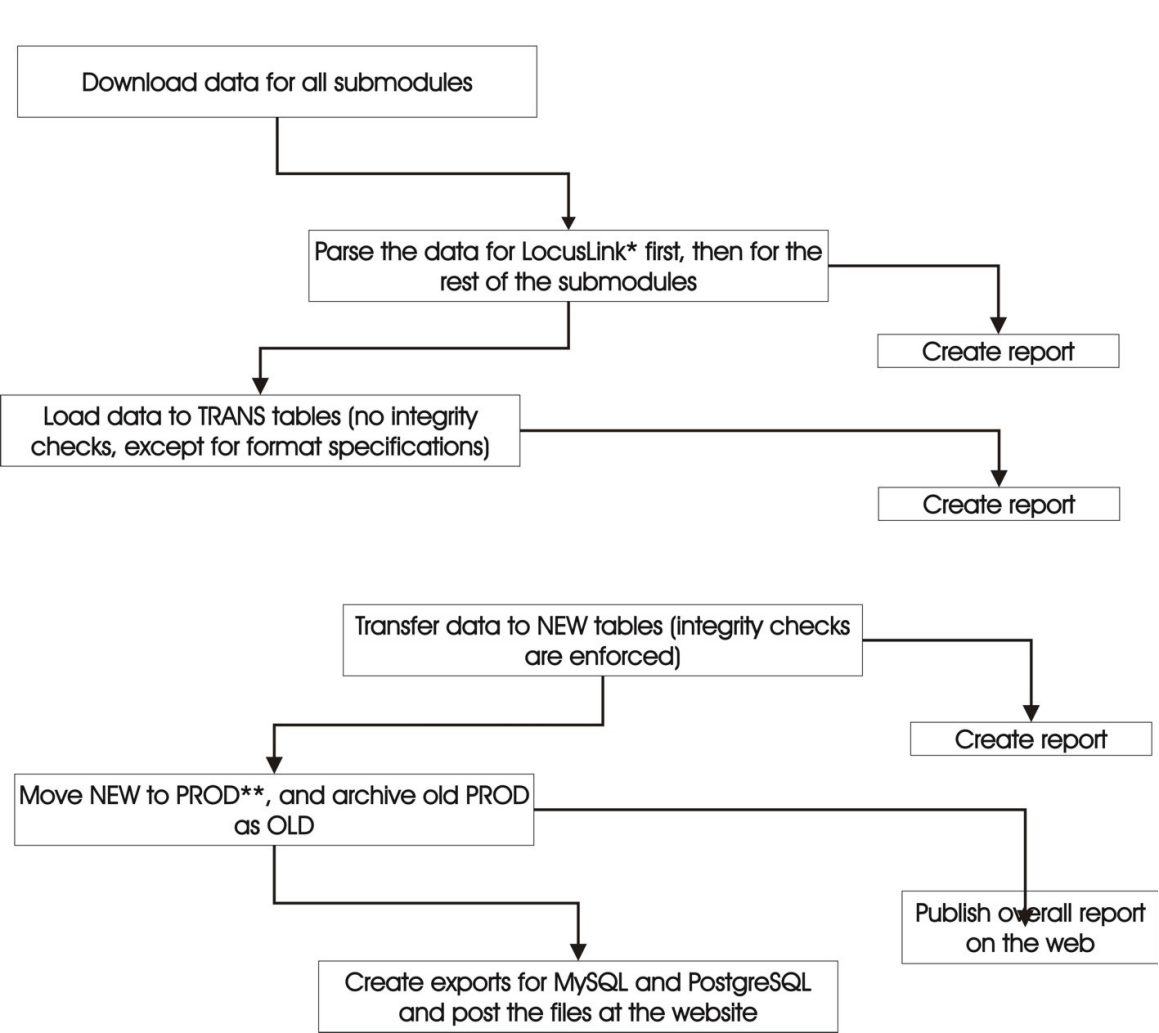
A workflow schema of GeneKeyDB creation and export to other RDBMS. *LocusLink is parsed first as other sub-modules depend on it with respect to the central key of the database. PROD refers to the current production stage database.

The database is created using Oracle and the parsers are written in Perl. Bioperl and ENSEMBL API are also used to create some of the tables. A brief overview of the database creation process is shown in Figure 2. Though we do not cleanse the source data, there are natural restrictions that should be enforced. For example no RefSeq accession number can be mapped to two LocusLink identifiers, no ensembl gene stable identifier can be mapped to more than one LocusLink identifiers, and the combination of the gene symbol and organism name should be unique.

LocusLink tables are derived through parsing LocusLink flat files and inferring additional data such as absolute chromosome coordinates, calculated by joining NCBI contig builds (see supplemental data at GeneKeyDB website [11]). These tables describe fundamental information about a particular gene: name, description, associated accession numbers, chromosome location, suggested function, comparative map information among other variables. ENSEMBL originating information also occupies a significant part of the database. It holds the relationship between LocusLink identifiers and ENSEMBL, the original tables used to create this correlation, and additional inferred data such as absolute chromosome coordinates. The CGAP data is parsed into several tables, two of which hold the expression and cDNA library data, while the rest hold functional annotation. These tables are created and updated automatically twice per week, except Affymetrix data, which must be downloaded manually from a password protected area. The update takes approximately 12 hours, including download, parsing and loading. Each sub module can be processed in a distributed fashion (after the central module, Locuslink is parsed), thus significantly reducing the time needed for the update. This allows new modules to be added without noticeable impact over the time that the update process will take This allows us to user more than one processor, for example At the conclusion of the process, new data is exported both as comma delimited and tab delimited files along with the create statements for Oracle, mysql and PostgreSQL RDBMS.

It is important to note that no extensive cleansing of the data is performed during the database creation and update process. This allows automatic updates and eliminates some well known problems created by data cleansing [12]. At the same time, the database automates the creation of views that display the best solution to possible conflicting entries, usually derived from different sources. An example of such conflicts are chromosome coordinates (UCSC, NCBI, Ensembl) and groups of orthologues (Homologene, MGI).

Currently we are migrating from LocusLink data to Entrez Gene data as it becomes available from NCBI.

## Utility and discussion

GeneKeyDB is primarily used for automating the integration of large data sets with a variety of computational tools that examine secondary gene relationships, such as the analysis of gene regulatory networks and their evolution. Current tools heavily relying on GeneKeyDB include the BSA pipeline (manuscript in preparation), GoTree machine and other utilities available on our web site. GoTree and Webgestalt [13] for example, rely on GeneKeyDB to generate gene ontology relationships.

The database was constructed, following some of the guidelines and logic described by Waterman et al. [14]. GeneKeyDB can be used to answer similar to or even more complex question than the ones described in the same work as shown by the following set of examples (more details available from GeneKeyDB website):

1. Define a set of candidate genes, based on the genome localization and tissue expression pattern[15]

2. Get all genes expressed in a specific tissue that share a conserved protein domain[16].

3. Get a set of orthologues to a group of genes (defined by the input) and obtain their genome coordinates [17]

These examples are designed to help formulate sets of genes, sharing some functional, sequence or expression similarity. These results are not permanent as the information differs between releases. The times for executing the queries was less than 30 s. per each one and have been produced under Oracle 9.0i database on Sparc Ultra-4, SunOS 5.8.

While such queries are complex, GeneKeyDB's lightweight nature allows its conversion to popular databases such as Microsoft Access and FileMaker Pro, where graphical user interfaces allow for the creation of queries with only a rudimentary knowledge of SQL. Even though Microsoft Access and FileMaker have limited capabilities compared to Oracle, MySQL or PostgreSQL, they succeeded in handling GeneKeyDB.

## Conclusion

The development of more exhaustive high-throughput experimental procedures has led to the accumulation of abundant biological data resources. As a consequence, the LocusLink approach of one-gene-at-a-time is an insufficient method to properly mine the data and therefore, analysis of sets of genes is more productive. Additionally, converting between multiple database identifiers is still a challenge as there is no current method to uniquely identify the same gene across multiple databases. Therefore a data mining environment that can synchronize multiple sources and provide general annotation information is going to be beneficial for comparing and using results originating from different experimental groups. One excellent existing resource is EnsMART[5], but its complexity and size can be overwhelming, especially if system integration is needed. GeneKeyDB can be used to address the same issues, but may be run locally and is roughly 20 times smaller. At approximately 1 GB, it is easily integrated with new and existing computational tools. For the same reason, GeneKeyDB can be used to support distributed analysis for purposes of genome annotation, regulatory network predictions, and other analysis pipelines. Another advantage is that any tool based on GeneKeyDB will rely on standard SQL to manipulate data instead of a proprietary code. On the other hand, GeneKeyDB could easily interact with BioSQL database[18], with GeneKeyDB providing the central keys and relations between them, while BioSQL can supply sequence data and additional annotation also through standard SQL. This creates a system that is both very flexible and powerful.

Compared to the most similar database, DRAGON, GeneKeyDB has significantly more keys and relationships (CGAP, LocusLink, MGI, Homologene, Affymetrix identifiers, etc), where DRAGON has only some PFAM, Unigene and Incyte data as TREMBL, Transfac and Interpro data is not implemented yet in this database (see DRAGON database website [19]).

Through the GeneKeyDB database, it is possible to bring together different data such as evolutionary (HomoloGene, MGI comparative map), expression (CGAP), physical location and functional annotation (GO) in a high throughput fashion, making this resource valuable both to experimental and bioinformatics groups. Though the database is most useful when installed locally, some web based functionality also exists. The most significant function that this system provides is a small lightweight database that can enable analysis tools and local database to have the flexible functions of database queries about genes, gene products, and sets of genes in the course of their large-scale analysis.

Current and future development includes improvement to the MySQL/PostgreSQL export, migration toward Entrez Gene (done gradually as new data becomes available at the FTP site), test integration with BioSQL, etc. We intend to convert GeneKeyDB to an open source project and share our parsers with other relevant open source project such as BioPerl wherever appropriate. We expect this step to help GeneKeyDB maintenance.

## Availability and requirements

SQL access (through web), Oracle, PostgreSQL and MySQL downloads are available from .

Project Name: GeneKeyDB

Project Homepage: 

Operating System: Platform independent, tested under Linux and Unix

Other Requirements: RDBMS, preferably Oracle, MySQL or PostgreSQL

License: GNU GPL

Any Restrictions to use by non-academics: License needed

## List of abbreviations

RDBMS- relational database management system

SQL- simple query language

UIs- user interfaces

APIs- application programming interfaces

GOTM- GO Tree Machine

GRIF- Gene Reference Into Function

## Authors' contributions

SK drafted the manuscript and is currently implementing the newest features in GeneKey. SK, EB and BZ are using aspects of GeneKeyDB to extend the data mining of gene sets. DS, SK, BZ, XP and EB developed the GeneKeyDB database. JS guided and coordinated execution of the project. All authors read and approved the final manuscript.

## Figures and Tables

**Table 1:** Comparison of different databases, which could be used to annotate and analyze large-scale biological data. Simplified joins refers to the availability of a central key and the ability to join tables through simple queries. Remote access column refers only to a machine access to a database server.

Database	SQL	Web interface	Simplified joins	^1^Remote access	Automatic updates provided	Type	Structure available as	Modular design	Download
GeneKeyDB	Yes	^2^Indirect	Yes	No	Yes	Oracle	Oracle, mysql, postgresql	Yes	Yes
LocusLink (Entrez Gene)	No	Yes	Na	Na	No	Flat file(s)	Flat file only	Na	Yes
RefSeq	No	Yes	Na	Na	No	Flat file	Flat file only	Na	Yes
Ensembl (core databases)	Yes	Yes	No	Yes	No	Mysql	Mysql	No	Yes
EnsMART	Yes	Yes	Yes	Yes	No	Mysql	Mysql	No	Yes
Dragon	Yes	Yes	No	No	No	Mysql	Flat file only	No	Data files only
HomoloGene	No	Yes	Na	Na	No	Flat files, XML	XML	No	Yes

^1^Refers only to machine access to the relational databases.

^2^GeneKeyDB serves as a data mining environment to different tools, therefore these tools are could also be considered a part of the interface layer.

**Table 2:** Summary of the attributes provided by each source

Sub module	Source	Attributes
CGAP	CGAP	Expression data, LocusLink ID to Unigene and Genbank Accession; Unigene to KEGG/GO/Biocarta
UCSC	UCSC RefGene	LocusLink ID to chromosome coordinates and exon structure
LocusLink	LocusLink (Entrez Gene)	Locuslink to Genbank accesion number, RefSeq Accession numbers, Gene descriptions (symbol, name, etc.), GRIF, Pubmed, OMIM, CDD, map location,
MGI	MGI comparative map	Homology data
Homologene	Homologene	Homology data
Ensembl	Ensembl	LocusLink ID to Ensebml Gene and Transcript Stable IDs, Contig data
